# Determining reference ranges and sample sizes in parallel-group studies

**DOI:** 10.1371/journal.pone.0278447

**Published:** 2022-11-30

**Authors:** Gwowen Shieh

**Affiliations:** Department of Management Science, National Yang Ming Chiao Tung University, Hsinchu, Taiwan; Bu-Ali Sina University: Bu Ali Sina University, ISLAMIC REPUBLIC OF IRAN

## Abstract

**Background and objectives:**

Reference ranges are widely used to locate the major range of the target probability distribution. When future measurements fall outside the reference range, they are classified as atypical and require further investigation. The fundamental principles and statistical properties of reference ranges are closely related to those of tolerance interval procedures. Existing investigations of reference ranges and tolerance intervals mainly devoted to the primitive cases of one- and paired-sample designs. Although reference ranges hold considerable promise for parallel group designs, the corresponding methodological and computational issues for determining reference limits and sample sizes have not been adequately addressed.

**Methods:**

This paper describes a complete collection of one- and two-sided reference ranges for assessing measurement differences in parallel-group studies that assume variance homogeneity.

**Results:**

The problem of sample size determination for precise reference ranges is also examined under the expected half-width and assurance probability considerations. Unlike the current methods, the suggested sample size criteria explicitly accommodate desired interval width in precise interval estimation.

**Conclusions:**

Theoretical examinations and empirical assessments are presented to validate the usefulness of the proposed reference range and sample size procedures. To enhance the usages of the recommended techniques in practical applications, computer programs are developed for efficient calculation and exact analysis. A real data example regarding tablet absorption rate and extent is presented to illustrate the suggested assessments between two drug formulations.

## Introduction

Reference ranges are commonly used as a decision-making tool in medical practice. The conventional confidence intervals provide the potential bounds for a single-value mean parameter. Yet the reference ranges give distinctly different implications in quantifying a plausible range for the major proportion of a distribution. The reference limits of a constructed reference range comprise a substantial part of the population measurements with the designated confidence level. Specifically, the reference limits of 95% proportion encompass the 2.5th percentile and 97.5th percentile for the target population. The main use of reference ranges is to classify future observations, and the measurements fall outside the reference range are considered as atypical and necessitate additional evaluations. Practical guidelines and general discussions can be found in Harris and Boyd [1], Horn and Pesce [2], and Horowitz et al. [3]. Moreover, important applications and principles were presented in Ceriotti, Hinzmann, and Panteghini [4], Geffre et al. [5], Henny and Petersen [6], Jensen and Kjelgaard-Hansen [7], Klee et al. [8], Siest et al. [9], and Wellek et al. [10].

It is noteworthy that reference ranges to estimate a designated proportion of a population are statistically equivalent to tolerance intervals to contain at least a specified proportion of a distribution considered by Wald and Wolfowitz [11]. The inferential properties of tolerance intervals are readily applicable to clarify the utility of reference ranges. Technical discussions of tolerance interval estimation are available in the excellent texts by Krishnamoorthy and Mathew [12] and Meeker, Hahn, and Escobar [13]. Methodological update and related comparisons of tolerance intervals are available in the recent studies of Francq, Berger, and Boachie [14], Liu, Bretz, and Cortina-Borja [15], and Roshan et al. [16]. Although the applications of reference ranges mainly concentrate in laboratory medicine and industrial engineering, the concept and analysis are pertinent to comparative studies across virtually all disciplines. Moreover, the functional versatility of reference ranges is also extended to evaluate exchangeability or switchability in method comparisons. Related applications of reference range or tolerance interval procedures for agreement assessments were demonstrated in Francq et al. [13], Gerke [17], Jan and Shieh [18], and Shieh [19].

Sample size determination for precise tolerance intervals has long been recognized in Wilks [20] for distribution-free tolerance limits. This practical problem has attracted considerable attention in the statistical literature. A common approach is to insure there is a small probability that the derived tolerance interval will exceed the desired proportion by a small margin. Due to the computational complexity, several approximations were considered in the early work by, among others, Faulkenberry and Weeks [21], Faulkenberry and Daly [22], and Guenther [23]. Instead, Odeh, Chou, and Owen [24] presented exact calculations for tolerance intervals to control the major proportion of a distribution. Moreover, Chou and Mee [25] described sample size procedure for tolerance intervals to control equal tail probability of a distribution. These procedures require specifying two key factors of a small probability and a marginal proportion for sample size determination. Note that the selections of appropriate specifications and their impact on the resulting tolerance intervals are not always clear and interpretable. Accordingly, Jennen-Steinmetz and Wellek [26] and Young et al [27] proposed alternative sample size methods for reference range and tolerance interval studies.

The current literature of reference range procedures and sample size determinations mainly focuses on the simple cases of one- and paired-sample designs. The principles have been generalized to distinct scenarios in various research fields. Although the applications of reference ranges are inevitably important for parallel group designs, the corresponding methodological and computational issues for determining reference limits and sample sizes have not been adequately addressed. Consequently, no single resource appropriately describes the technical and computational aspects of reference ranges and sample size calculations for parallel group designs under variance homogeneity. Appropriate use of reference ranges is likely hampered by an absence of knowledge of their properties. Both researchers and practitioners may appreciate a concise document of the primary development. On the other hand, the analytic complexity requires computer algorithms to compute the exact reference limits and optimal sample sizes under various design configurations. The use of reference range and sample size techniques can be significantly impeded by a lack of specialized computer software.

To complement the findings in the literature, the current study has three major goals. First, both one- and two-sided reference ranges are described for assessing measurement differences in parallel-group studies under homogeneous variance conditions. They encompass two types of reference ranges to control the major proportion and to control equal tail proportions of the measurement differences. In the process, approximations are also presented and examined to justify the advantages of the recommended exact approaches. Second, the prevalent criterion for determining optimal sample size requires that the probability be small that the tolerance interval covers too large a proportion of the sampled population. It is sensible to apply a rule that directly controls the potential width of a tolerance interval. Sample size procedures for precise reference ranges are proposed under the expected half-width and assurance probability considerations. Third, the described methods for parallel group designs are not available in current software packages. To alleviate the theoretical complexity and computational demand, computer algorithms are developed for the suggested reference range and sample size calculations. Numerical illustrations are also provided to demonstrate the advancement and usage of the exact approaches and accompanying software programs.

## Methods

### Reference ranges

Consider independent random samples *X*_*ij*_ from two normal populations:

$$X_{ij}\sim N(\mu_{i},\sigma^{2}),$$ (1)

where μ_*i*_, σ^2^ are unknown parameters, *j* = 1, …, *N*_*i*_, and *i* = 1 and 2. For detecting the group effect μ_*D*_ = μ_1_ − μ_2_ in terms of the hypothesis H_0_: μ_*D*_ = 0 versus H_1_: μ_*D*_ ≠ 0, the usual two-sample *t* statistic has the form

$$T=\frac{{\bar{X}}_{1}-{\bar{X}}_{2}}{{\left(S^{2}/M\right)}^{1/2}},$$

where ${\bar{X}}_{1}=\sum_{j=1}^{N_{1}}\;X_{1j}/N_{1},\;{\bar{X}}_{2}=\sum_{j=1}^{N_{2}}\;X_{2j}/N_{2}$, *M* = 1/(1/*N*_1_ + 1/*N*_2_), $S^{2}=\{\left(N_{1}-\;1\right)S_{1}^{2}+(N_{2}-1)S_{2}^{2}\}/\nu$, $S_{1}^{2}=\sum_{j=1}^{N_{1}}{\left(X_{1j}-{\bar{X}}_{1}\right)}^{2}/\left(N_{1}-1\right)$, $S_{2}^{2}=\sum_{j=1}^{N_{2}}{\left(X_{2j}-{\bar{X}}_{2}\right)}^{2}/\left(N_{2}-1\right)$, and ν = *N*_1_ + *N*_2_ − 2.

Note that the difference *D* = *X*_1*j*_ − *X*_2*j*′_ between two measurements of the normal populations has the distribution

$$D\sim N(\mu_{D},\sigma_{D}^{2}),$$ (2)

where $\sigma_{D}^{2}-2\sigma^{\text{2}}$. Accordingly, the 100*p*th percentile of the normal distribution $N(\mu_{D},\sigma_{D}^{2})$ is denoted by θ_*p*_, where

$$\theta_{p}=\mu_{D}+z_{p}\sigma_{D}$$ (3)

and *z*_*p*_ is the (100·*p*)th percentile of the standard normal distribution *N*(0, 1) and 0 < *p* < 1.

#### One-sided reference ranges for a major proportion

To provide a reference range of the major proportion p* for the distribution of measurement difference *D*, a feasible approach is to consider the one-sided reference ranges for the percentile θ_p*_ = μ_*D*_ + *z*_p*_σ_*D*_ where 0 < p* < 1. Standard derivations show that

$$T^{*}=\frac{\;{\bar{X}}_{1}-{\bar{X}}_{2}-\theta_{p*}}{{\left(S^{2}/M\right)}^{1/2}}\sim t(\nu ,-{\left(2M\right)}^{1/2}z_{p*}),$$ (4)

where *t*(ν, −(2*M*)^1/2^*z*_p*_) is a noncentral *t* distribution with degrees of freedom ν and noncentrality parameter–(2*M*)^1/2^*z*_p*_. First, a 100(1 –α)% one-sided lower confidence interval of θ_p*_, with p* > 0.5, is expressed as *C*_*LP*_ = (–∞, *T*_*LPU*_) and $T_{LPU}={\bar{X}}_{1}\;-\;{\bar{X}}_{2}\;-\;t_{\alpha }(\nu ,\;-{\left(2M\right)}^{1/2}z_{p*}){\left(S^{2}/M\right)}^{1/2}$ where *t*_*α*_(*ν*, −(2*M*)^1/2^*z*_p*_) is the (100 α)th percentile of the distribution *t*(*ν*, −(2*M*)^1/2^*z*_p*_). The upper confidence limit *T*_*LPU*_ is conveniently expressed as

$$T_{LPU}={\bar{X}}_{1}-{\bar{X}}_{2}+\tau_{1-\alpha }{\left(S^{2}/M\right)}^{1/2},$$ (5)

where τ_1−*α*_ = *t*_1−*α*_(*ν*, (2*M*)^1/2^*z*_p*_) = *t*_*α*_(*ν*, −(2*M*)^1/2^*z*_p*_).

Second, a 100(1 –α)% one-sided upper confidence interval of θ_1−p*_ = μ_*D*_ − *z*_p*_σ_*D*_, with 1 –p* < 0.5, is denoted by *C*_*UP*_ = (*T*_*UPL*_, ∞) and the lower confidence limit *T*_*UPL*_ has the form

$$T_{UPL}={\bar{X}}_{1}-{\bar{X}}_{2}-\tau_{1-\alpha }{\left(S^{2}/M\right)}^{1/2}.$$ (6)

Note that the upper confidence limit *T*_*LPU*_ of θ_p*_ assures that $P\{P[D<T_{LPU}|({\bar{X}}_{1}-{\bar{X}}_{2},S^{2})]\geq p^{*}\}=1-\alpha$. Hence, an upper 100(1 –α)% confidence limit on the (100·p*)th percentile for p* > 0.5 is equivalent to an upper tolerance limit to exceed at least a proportion p* of the population with probability 1 –α. On the other hand, the lower confidence limit *T*_*UPL*_ of θ_1−p*_ guarantees that $P\{P[D<T_{UPL}|({\bar{X}}_{1}-{\bar{X}}_{2},S^{2})]\geq p^{*}\}=1-\alpha$. Thus, a lower 100(1 –α)% confidence limit on the 100(1 –p*)th percentile for 1 –p* < 0.5 is equivalent to a lower tolerance limit to be exceeded by at least a proportion p* of the population with probability 1 –α. As noted in Hahn [28,29], the one-sided reference ranges of percentiles are equivalent to the one-sided tolerance intervals for the designated proportion p* of population. Similar results were presented in Section 2.4.1 Krishnamoorthy and Mathew [12] when the variance ratio is known.

#### Two-sided reference ranges for a major proportion

A general form of two-sided reference ranges to control the major proportion p* for the target population of measurement difference *D* is expressed as

$$C_{MP}=(T_{MPL},T_{MPU}),$$ (7)

where $T_{MPL}={\bar{X}}_{1}-{\bar{X}}_{2}-g_{1-\alpha }{\left(S^{2}/M\right)}^{1/2}$, $T_{MPU}={\bar{X}}_{1}-{\bar{X}}_{2}-g_{1-\alpha }{\left(S^{2}/M\right)}^{1/2}$, and *g*_1−*α*_ is a designated value so that

$$P\{P[T_{MPL}<D<T_{MPU}|({\bar{X}}_{1}-{\bar{X}}_{2},S^{2})]\geq p^{*}\}=1-\alpha \text{.}$$ (8)

Following the detailed derivations in Appendix A1, the critical value *g*_1−*α*_ can be uniquely determined from

$$E_{Z}\{1-\Phi_{K}(K_{g})\}=1-\alpha ,$$ (9)

where the expectation *E*_*Z*_ is taken with respect to the standard normal distribution *Z*, *K*_*g*_ is given in Equation A2 in S1 File, and Φ_*K*_(·) is the cumulative distribution function of the chi-square random variable *K* ~ χ^2^(ν). Despite the critical value *g*_1−*α*_ does not have an explicit analytic form, the simplification in Eq 9 provides a particularly attractive formulation for computing the critical value and reference range. Related discussions for tolerance intervals of designated major proportions can be found in Krishnamoorthy, Lian and Mondal [30] when the variance ratio is known and unknown. However, they did not cover tolerance interval estimation for the central proportion with equal tail probabilities.

#### Two-sided reference ranges for the central proportion

The second type of reference ranges specialize on the central proportion p* and controls the equal tail proportions in terms of the paired percentiles {θ_*p*_, θ_1−*p*_} = {μ_*D*_ − *z*_*p*_σ_*D*_, μ_*D*_ + *z*_*p*_σ_*D*_} with *p* > 0.5 and p* = (2*p* − 1). Hence, it is desirable to find the 100(1 –α)% confidence interval

$$C_{ET}=(T_{ETL},T_{ETU})$$ (10)

to cover the central proportion p* of the distribution of measurement difference *D* where $T_{ETL}={\bar{X}}_{1}-{\bar{X}}_{2}-h_{1-\alpha }{\left(S^{2}/M\right)}^{1/2},$ $T_{ETU}={\bar{X}}_{1}-{\bar{X}}_{2}-h_{1-\alpha }{\left(S^{2}/M\right)}^{1/2},$ and *h*_1−*α*_ is a selected value so that

$$P\{T_{ETL}<\theta_{1-p}\;\text{and}\;\theta_{p}<T_{ETU}\}=1-\alpha \text{.}$$ (11)

It follows from the technical arguments in Appendix A2 that the critical value *h*_1−*α*_ can be uniquely computed from

$$E_{Z}\{1\;-\Phi_{K}(K_{h})\}=1-\alpha ,$$ (12)

where the expectation *E*_*Z*_ is taken with respect to the standard normal distribution *Z* and *K*_*h*_ is given in Equation A4 in S1 File. In general, the critical value *h*_1−*α*_ does not have a close-form expression. However, the expression in Eq 12 gives a computationally convenient formulation for obtaining the critical value and reference range.

Notably, the derived expressions for in Eqs 9 and 12 demonstrate the close resemblance between the confidence level appraisals for the control of major proportion and central proportion, respectively. It is believed that no previous attempt has made to give the unified and transparent formulations. Accordingly, the required calculations of the critical values *g*_1−*α*_ and *h*_1−*α*_ can be conducted with the embedded normal and chi-square distribution functions in popular software packages. The corresponding computer algorithms are developed for implementing the described computations. To quantify the desired range of distribution, the control of equal tails of the distribution is more stringent than the control of the sum of two tails of the distribution. When all the factors are identical, the resulting critical value *h*_1−*α*_ for the coverage of central proportion is larger than the counterpart *g*_1−*α*_ for the coverage of major proportion: *h*_1−*α*_ > *g*_1−*α*_. Therefor, the reference range *C*_*ET*_ is generally wider than the reference range *C*_*MP*_ under the identical model settings. The discrepancy between the different types of reference ranges in terms of interval widths and sample sizes will be illustrated in the subsequent analysis.

#### Approximations and numerical comparisons

The reference ranges of major proportion and central proportion require a specialized algorithm to compute the critical value for the designated settings of sample sizes (*N*_1_, *N*_2_), confidence level 1 –α, distribution proportion p*. It is temping to adopt simplified procedures with less computation. Similar to the simplification in Wald and Wolfowitz [11] for the tolerance intervals of major proportion in one-sample case, two approximations are presented here.

With the prescribed result *Z* ~ *N*(0, 1), it is clear that *E*[*Z*^2^] = 1. Thus, the noncentrality λ^2^ of $\chi_{p*}^{2}(1,\;\lambda^{2})$ has the approximation λ^2^ = *Z*^2^(2*M*) ≐ *E*[*Z*^2^]/(2*M*) = 1/(2*M*). The confidence level in Equation A2 in S1 File is simplified as *P*{*K* > *A*_*g*_} = 1 –α where $A_{g}=(2M\nu )\chi_{p*}^{2}(1,\;1/(2M))/{\hat{g}}_{1-\alpha }^{2}$. It follows from *K* ~ χ^2^(ν) that

$${\hat{g}}_{1-\alpha }={\left\{(2M\nu )\chi_{p*}^{2}(1,\;1/(2M))/\chi_{\alpha }^{2}(\nu )\right\}}^{1/2},$$ (13)

where $\chi_{\alpha }^{2}(\nu )$ is the (100·α)th percentile of $\chi_{\alpha }^{2}(\nu )$. Accordingly, a approximate two-sided tolerance interval to control the major proportion p* for the target population of measurement difference *D* is denoted by

$$C_{AMP}=(T_{AMPL},T_{AMPU}),$$ (14)

where $T_{AMPL}={\bar{X}}_{1}-{\bar{X}}_{2}-{\hat{g}}_{1-\alpha }{\left(S^{2}/M\right)}^{1/2}$ and $T_{AMPU}={\bar{X}}_{1}-{\bar{X}}_{2}-{\hat{g}}_{1-\alpha }{\left(S^{2}/M\right)}^{1/2}$.

Moreover, the same simplification can also be applied to the critical values of central proportion. Specifically, with the approximation |*Z*| = (*Z*^2^)^1/2^ ≐ *E*[(*Z*^2^)^1/2^] = (2/π)^1/2^, the evaluation in Equation A4 in S1 File is rewritten as *P*{*K* > *A*_*h*_} = 1 –α where $A_{h}=(2M\nu ){\left\{1/{\left(\pi M\right)}^{1/2}+z_{p}\right\}}^{2}/{\hat{h}}_{1-\alpha }^{2}$. With *K* ~ χ^2^(ν), the approximate critical value is

$${\hat{h}}_{1-\alpha }={\left\{(2M\nu ){\left\{1/{\left(\pi M\right)}^{1/2}+z_{p}\right\}}^{2}/\chi_{\alpha }^{2}(\nu )\right\}}^{1/2}.$$ (15)

Thus, a approximate two-sided tolerance interval to control the central proportion p* for the target population of measurement difference *D* is

$$C_{AET}=(T_{AETL},T_{AETU}),$$ (16)

where $T_{AETL}={\bar{X}}_{1}-{\bar{X}}_{2}-{\hat{h}}_{1-\alpha }{\left(S^{2}/M\right)}^{1/2}$ and $T_{AETU}={\bar{X}}_{1}-{\bar{X}}_{2}-{\hat{h}}_{1-\alpha }{\left(S^{2}/M\right)}^{1/2}$.

To demonstrate the properties of the exact and approximate reference ranges {*C*_*MP*_, *C*_*AMP*_, *C*_*ET*_, *C*_*AET*_}, simulation studies of 10,000 iterations were conducted to examine their coverage performance under various situations. Note that the coverage performance of the exact and approximate reference ranges is a function of sample sizes, distribution proportion, and confidence level. Table 1 presents the simulated coverage probabilities and errors for the 90% two-sided reference ranges of the distribution proportion 0.80. A total of 12 combined balance and unbalance designs (*N*_1_, *N*_2_) are considered where *N*_1_ = 5, 10, and 25, and the other sample size *N*_2_ = *rN*_1_ with *r* = 1, 2, 5, and 10. On the other hand, the accuracy of the examined coverage does not depend on the population parameters μ_*D*_ and σ^2^. Without loss of generality, the underlying parameters are set as μ_*D*_ = 0 and σ^2^ = 1.

**Table 1:** The simulated coverage probabilities and errors for the 90% two-sided reference intervals of the distribution proportion 0.80.

		Major proportion				Equal tails			
		Exact method *C*_*MP*_		Approximation *C*_*AMP*_		Exact method *C*_*ET*_		Approximation *C*_*AET*_	
*N*_1_	*N*_2_	Simulated coverage	Difference	Simulated coverage	Difference	Simulated coverage	Difference	Simulated coverage	Difference
5	5	0.8969	–0.0031	0.8901	–0.0099	0.8991	–0.0009	0.9105	0.0105
	10	0.8988	–0.0012	0.8865	–0.0135	0.8988	–0.0012	0.9053	0.0053
	25	0.8997	–0.0003	0.8803	–0.0197	0.8985	–0.0015	0.8951	–0.0049
	50	0.8968	–0.0032	0.8655	–0.0345	0.8991	–0.0009	0.8743	–0.0257
10	10	0.8954	–0.0046	0.8864	–0.0136	0.8967	–0.0033	0.9034	0.0034
	20	0.8997	–0.0013	0.8887	–0.0113	0.8968	–0.0032	0.8995	–0.0005
	50	0.8986	–0.0014	0.8830	–0.0170	0.8975	–0.0025	0.8860	–0.0140
	100	0.9038	0.0038	0.8807	–0.0193	0.9025	0.0025	0.8697	–0.0303
25	25	0.8951	–0.0049	0.8882	–0.0118	0.8964	–0.0036	0.8976	–0.0024
	50	0.8978	–0.0022	0.8908	–0.0092	0.8972	–0.0028	0.8951	–0.0049
	125	0.9037	0.0037	0.8956	–0.0044	0.9037	0.0037	0.8859	–0.0141
	250	0.9009	0.0009	0.8892	–0.0108	0.9010	0.0010	0.8630	–0.0370

The results in Table 1 show that the approximate interval *C*_*AMP*_ is sensitive to unbalance designs especially when the sample sizes are small, such as those with *N*_1_ = 5 and 10. The reported errors are –0.0345, –0.0193 and –0.0108 for the cases of (*N*_1_, *N*_2_) = (5, 50), (10, 100), and (25, 250), respectively. The other approximate interval *C*_*AET*_ also reveals the same disadvantage and the problematic situation continues to deteriorate as the sample size *N*_1_ increases from 5 to 25. Specifically, the resulting errors are –0.0257, –0.0303, and –0.0370 for the unbalance cases (*N*_1_, *N*_2_) = (5, 50), (10, 100), and (25, 250), respectively.

In contrast, the exact reference ranges *C*_*MP*_ and *C*_*ET*_ maintain excellent coverage performance for all the configurations. These model configurations do not really cover a wide range of scenarios in parallel group trial. However, these findings discern the essential behavior of the approximate procedures that may not be revealed under other settings. The two simple modifications *C*_*AMP*_ and *C*_*AET*_ provide computational shortcuts and reasonable approximations, but their accuracy is vulnerable to small magnitude and unbalance structure of sample sizes. Moreover, the calculations of approximate intervals may still require the aid of computer software. The additional computational effort of the exact approaches seems inconsequential if the corresponding computer algorithms are available. In short, the exact reference ranges can be recommended for general use in comparative studies.

## Results

### Sample size determinations

Within the context of research design, a subject of great importance is to determine the optimal sample sizes so that the resulting confidence interval will meet the designated precision requirement. The existing sample size procedures for reference ranges or tolerance intervals mainly focus on the one-sample or paired-sample frameworks, such as Meeker, Hahn, and Escobar [13] for two-sided tolerance intervals, among others. Also, the common criterion constricts the tail proportions will not be too small at a given confidence level. As an alternative, the control of the expected width and the assurance probability of the width within a designated bound of Beal [31] and Kupper and Hafner [32] are considered and demonstrated here for precision evaluations of reference ranges.

#### Precision of the one-sided reference ranges for a major proportion

The one-sided reference ranges *C*_*LP*_ = (–∞, *T*_*LPU*_) and *C*_*UP*_ = (*T*_*UPL*_, ∞) for a major proportion have an open end. Due to the unbounded limits, their precision levels rely on the finite limits *T*_*LPU*_ and *T*_*UPL*_. It is an intuitive and feasible way to measure their precision through the half-width between ${\bar{X}}_{1}-{\bar{X}}_{2}$ and *T*_*LPU*_ for *C*_*LP*_, and the width between *T*_*UPL*_ and ${\bar{X}}_{1}-{\bar{X}}_{2}$ for *C*_*UP*_. In both cases, the half-width is denoted by *H*_*τ*_ = τ_1−*α*_(*S*^2^/*M*)^1/2^.

Notably, it is desired to calculate the least sample size such that the expected half-width of the one-sided reference range is within the given threshold:

$$E[H_{\tau }]<\eta ,$$ (17)

where η (> 0) is a constant. In addition, one may compute the minimum sample size needed to guarantee, with a given assurance probability, that the suggested half-width of a one-sided reference range will not exceed the planned value:

$$P(H_{\tau }\leq \eta )\geq 1-\gamma ,$$ (18)

where 1 –γ ∈ (0, 1) is the specified assurance level and η (> 0) is a constant.

It can be shown that the expected half-width η_*τ*_ = *E*[*H*_*τ*_] is

$$\eta_{\tau }=(\tau_{1-\alpha }\sigma )/(uM^{1/2}),$$ (19)

where *u* = (ν/2)^1/2^Γ(ν/2)/Γ{(ν + 1)/2} is an adjusting factor so that *E*[*uS*] = σ and ν = *N*_1_ + *N*_2_ − 2. Moreover, the probability of half-width π_*τ*_ = *P*(*H*_*τ*_ ≤ η) is

$$\pi_{\tau }=\Phi_{K}(K_{\tau }),$$ (20)

where $K_{\tau }=(\nu M\eta^{\text{2}})/(\tau_{1-\alpha }^{2}\sigma^{2})$.

#### Precision of the two-sided reference ranges for a major proportion

The half-width of the two-sided reference range *C*_*MP*_ for a major proportion is *H*_*g*_ = (*T*_*MPU*_−*T*_*MPL*_)/2 = *g*_1−*α*_(*S*^2^/*M*)^1/2^. Thus, the expected half-width η_*g*_ = *E*[*H*_*g*_] is readily obtained as

$$\eta_{g}=(g_{1-\alpha }\sigma )/(uM^{1/2}).$$ (21)

Also, the probability of interval half-width π_*g*_ = *P*(*H*_*g*_ ≤ η) has the form

$$\pi_{g}=\Phi_{K}(K_{g}),$$ (22)

where $K_{g}=(\nu M\eta^{\text{2}})/(g_{1-\alpha }^{2}\sigma^{2})$.

#### Precision of the two-sided reference ranges for the central proportion

For the two-sided reference range *C*_*MP*_ of a central proportion, the half-width is *H*_*h*_ = (*T*_*ETU*_−*T*_*ETL*_)/2 = *h*_1−*α*_(*S*^2^/*M*)^1/2^. Accordingly, the associated expected half-width η_*h*_ = *E*[*H*_*h*_] is immediately derived as

$$\eta_{h}=(h_{1-\alpha }\sigma )/(uM^{1/2}).$$ (23)

In this case, the probability of interval half-width π_*h*_ = *P*(*H*_*h*_ ≤ η) has the form

$$\pi_{h}=\Phi_{K}(K_{h}),$$ (24)

where $K_{h}=(\nu M\eta^{\text{2}})/(h_{1-\alpha }^{2}\sigma^{2})$.

#### Sample size requirements

The precision quantities {η_*τ*_, η_*g*_, η_*h*_} and {π_*τ*_, π_*g*_, π_*h*_} are a monotone function of sample sizes (*N*_1_, *N*_2_) for fixed values of confidence level 1 –α, desired proportion p*, and standard deviation σ. However, the interval precision does not depend on the mean difference μ_*D*_. With the selected measure, the sample sizes needed to attain the specified precision can be found with a simple incremental search. Note that the critical values {τ_1−*α*_, *g*_1−*α*_, *h*_1−*α*_} are functions of sample sizes (*N*_1_, *N*_2_). In the iteration search, they need to be recalculated because the actual magnitude varies the sample sizes in each of the iterative processes. Accordingly, supplemental computer programs are presented to facilitate the required computations.

The features and differences of the suggested sample size procedures for precise reference range estimation are demonstrated with numerical assessments under the expected width and assurance probability criteria for two confidence levels 1 –α = 0.90 and 0.95. The selected three thresholds of expected half-width are η = 2.5, 3.0, and 3.5. For assurance evaluation, the designated assurance levels are 1 –γ = 0.8 and 0.9 combined with η = 2.5, 3.0, and 3.5. Moreover, the parameter configurations are fixed as μ_*D*_ = 0, σ^2^ = 1, and p* = 0.90 throughout the empirical appraisal. These configurations are chosen to reflect the potential discrepancy among necessary sample sizes of different reference ranges and precision configurations. The first step of the numerical illustration is to compute the required sample sizes for achieving the specified precision under balance designs and unbalance designs with *r* = *N*_2_/*N*_1_ = 1, 2, 5, and 10. The computed sample sizes are presented in Tables 2–7 for the expected half-width and assurance probability, respectively.

**Table 2:** The computed sample size, simulated half-width and errors for the 90% reference intervals of the distribution proportion 0.90.

	One-sided CI *C*_*LP*_ and *C*_*UP*_			Two-sided CI *C*_*MP*_			Two-sided CI *C*_*ET*_		
η	(*N*_1_, *N*_2_)	Simulated half-width	Difference	(*N*_1_, *N*_2_)	Simulated half-width	Difference	(*N*_1_, *N*_2_)	Simulated half-width	Difference
2.5	(13, 13)	2.4896	–0.0040	(100, 100)	2.4977	–0.0019	(317, 317)	2.4995	–0.0003
	(10, 20)	2.4597	–0.0032	(68, 136)	2.4999	0.0004	(227, 454)	2.4991	–0.0005
	(7, 35)	2.4469	–0.0011	(38, 190)	2.4969	–0.0012	(154, 770)	2.5003	0.0004
	(5, 50)	2.4837	–0.0047	(24, 240)	2.4983	–0.0012	(124, 1240)	2.4997	–0.0002
3.0	(6, 6)	2.9263	–0.0031	(13, 13)	2.9813	0.0038	(26, 26)	2.9900	0.0023
	(4, 8)	2.9628	–0.0050	(9, 18)	2.9796	0.0038	(18, 36)	2.9944	–0.0018
	(3, 15)	2.8435	–0.0039	(6, 30)	2.9258	0.0048	(12, 60)	2.9855	–0.0034
	(3, 30)	2.7060	0.0017	(4, 40)	2.9415	–0.0020	(10, 100)	2.9613	–0.0025
3.5	(4, 4)	3.3426	0.0003	(7, 7)	3.3832	–0.0090	(11, 11)	3.4409	0.0048
	(3, 6)	3.2396	–0.0041	(5, 10)	3.3660	0.0050	(8, 16)	3.4201	0.0091
	(3, 15)	2.8511	0.0037	(3, 15)	3.3430	–0.0052	(5, 25)	3.4107	–0.0077
	(3, 30)	2.7063	0.0020	(3, 30)	3.0945	–0.0029	(4, 40)	3.3779	0.0009

**Table 3:** The computed sample size, simulated half-width and errors for the 95% reference intervals of the distribution proportion 0.90.

	One-sided CI *C*_*LP*_ and *C*_*UP*_			Two-sided CI *C*_*MP*_			Two-sided CI *C*_*ET*_		
η	(*N*_1_, *N*_2_)	Simulated half-width	Difference	(*N*_1_, *N*_2_)	Simulated half-width	Difference	(*N*_1_, *N*_2_)	Simulated half-width	Difference
2.5	(21, 21)	2.4883	–0.0050	(154, 154)	2.4985	–0.0014	(451, 451)	2.4999	–0.0001
	(15, 30)	2.4895	–0.0027	(104, 208)	2.4996	–0.0003	(323, 646)	2.4999	0.0001
	(10, 50)	2.4947	–0.0018	(56, 280)	2.4996	0.0000	(219, 1095)	2.5002	0.0004
	(8, 80)	2.4972	–0.0014	(35, 350)	2.4972	–0.0014	(177, 1770)	2.4994	–0.0001
3	(9, 9)	2.9515	–0.0087	(18, 18)	2.9939	0.0003	(36, 36)	2.9979	0.0001
	(7, 14)	2.8844	–0.0031	(13, 26)	2.9746	0.0046	(26, 52)	2.9906	0.0010
	(4, 20)	2.9716	0.0010	(8, 40)	2.9441	–0.0006	(17, 85)	2.9877	–0.0013
	(3, 30)	2.9800	–0.0048	(5, 50)	3.0002	0.0049	(13, 130)	2.9906	–0.0032
3.5	(6, 6)	3.3451	0.0002	(9, 9)	3.4729	0.0058	(15, 15)	3.4573	–0.0018
	(4, 8)	3.3950	–0.0009	(6, 12)	3.4906	–0.0066	(10, 20)	3.4902	–0.0053
	(3, 15)	3.1958	–0.0033	(4, 20)	3.3800	0.0004	(7, 35)	3.4217	–0.0006
	(3, 30)	2.9851	0.0003	(3, 30)	3.3287	0.0066	(5, 50)	3.4478	–0.0042

**Table 4:** The computed sample size, simulated assurance probability and errors for the 90% reference intervals of the distribution proportion 0.90 with assurance probability 0.80.

	One-sided CI *C*_*LP*_ and *C*_*UP*_			Two-sided CI *C*_*MP*_			Two-sided CI *C*_*ET*_		
η	(*N*_1_, *N*_2_)	Simulated assurance probability	Difference	(*N*_1_, *N*_2_)	Simulated assurance probability	Difference	(*N*_1_, *N*_2_)	Simulated assurance probability	Difference
2.5	(24, 24)	0.8123	0.0041	(236, 236)	0.8049	0.0036	(557, 557)	0.7980	–0.0021
	(17, 34)	0.8105	–0.0017	(159, 318)	0.8040	0.020	(392, 784)	0.8037	0.0035
	(11, 55)	0.8209	–0.0033	(83, 415)	0.8042	0.0025	(250, 1250)	0.8117	0.0101
	(8, 80)	0.8267	0.0108	(49, 490)	0.8028	0.0013	(187, 1870)	0.8022	–0.0002
3.0	(10, 10)	0.8057	–0.0082	(24, 24)	0.8114	0.0006	(44, 44)	0.7963	–0.0042
	(7, 14)	0.8194	0.0029	(16, 32)	0.8038	0.0021	(31, 62)	0.8030	–0.0011
	(5, 25)	0.8822	–0.0033	(9, 45)	0.7986	–0.0045	(20, 100)	0.8229	–0.0012
	(3, 30)	0.7997	–0.0060	(6, 60)	0.8128	–0.0013	(14, 140)	0.8040	–0.0001
3.5	(6, 6)	0.8132	0.0062	(11, 11)	0.8166	0.0020	(18, 18)	0.8251	0.0019
	(5, 10)	0.8901	–0.0005	(8, 16)	0.8466	0.0040	(12, 24)	0.8048	0.0025
	(5, 25)	0.9947	0.0002	(5, 25)	0.8863	0.0004	(8, 40)	0.8490	–0.0010
	(3, 30)	0.9859	–0.0011	(3, 30)	0.8478	0.0023	(6, 60)	0.8814	–0.0007

**Table 5:** The computed sample size, simulated assurance probability and errors for the 90% reference intervals of the distribution proportion 0.90 with assurance probability 0.90.

	One-sided CI *C*_*LP*_ and *C*_*UP*_			Two-sided CI *C*_*MP*_			Two-sided CI *C*_*ET*_		
η	(*N*_1_, *N*_2_)	Simulated assurance probability	Difference	(*N*_1_, *N*_2_)	Simulated assurance probability	Difference	(*N*_1_, *N*_2_)	Simulated assurance probability	Difference
2.5	(31, 31)	0.9073	0.0012	(333, 333)	0.9060	0.0054	(710, 710)	0.8974	–0.0031
	(22, 44)	0.9102	–0.0008	(223, 446)	0.9040	0.0037	(496, 992)	0.9041	0.0041
	(14, 70)	0.9198	–0.0008	(115, 575)	0.9006	0.0005	(308, 1540)	0.8991	–0.0009
	(10, 100)	0.9187	0.0003	(67, 670)	0.9054	0.0032	(224, 2240)	0.9007	0.0001
3.0	(13, 13)	0.9147	–0.0010	(31, 31)	0.9034	0.0026	(56, 56)	0.8987	–0.0029
	(9, 18)	0.9157	0.0008	(21, 42)	0.8973	–0.0029	(39, 78)	0.9027	0.0012
	(6, 30)	0.9410	–0.0034	(12, 60)	0.9095	–0.0032	(24, 120)	0.9040	–0.0012
	(4, 40)	0.9297	–0.0019	(8, 80)	0.9305	0.0011	(17, 170)	0.9042	–0.0002
3.5	(8, 8)	0.9219	–0.0030	(14, 14)	0.9091	–0.0007	(22, 22)	0.9049	–0.0014
	(6, 12)	0.9497	0.0019	(10, 20)	0.9314	0.0073	(15, 30)	0.8990	–0.0013
	(5, 25)	0.9939	–0.0006	(6, 30)	0.9440	–0.0009	(9, 45)	0.8967	–0.0043
	(3, 30)	0.9860	–0.0010	(4, 40)	0.9530	–0.0018	(7, 70)	0.9394	–0.0019

**Table 6:** The computed sample size, simulated assurance probability and errors for the 95% reference intervals of the distribution proportion 0.90 with assurance probability 0.80.

	One-sided CI *C*_*LP*_ and *C*_*UP*_			Two-sided CI *C*_*MP*_			Two-sided CI *C*_*ET*_		
η	(*N*_1_, *N*_2_)	Simulated assurance probability	Difference	(*N*_1_, *N*_2_)	Simulated assurance probability	Difference	(*N*_1_, *N*_2_)	Simulated assurance probability	Difference
2.5	(35, 35)	0.8119	–0.0019	(319, 319)	0.8037	0.0030	(733, 733)	0.8007	0.0001
	(25, 50)	0.8234	0.0023	(214, 428)	0.8016	0.0011	(516, 1032)	0.8019	0.0017
	(16, 80)	0.8177	–0.0040	(111, 555)	0.8082	0.0067	(331, 1655)	0.7972	–0.0040
	(12, 120)	0.8244	0.0037	(65, 650)	0.8085	0.0050	(250, 2500)	0.8028	0.0003
3.0	(14, 14)	0.8078	0.0011	(31, 31)	0.8005	–0.0013	(58, 58)	0.8073	0.0052
	(10, 20)	0.8175	–0.0009	(22, 44)	0.8186	–0.0030	(41, 82)	0.8053	–0.0023
	(7, 35)	0.8857	0.0046	(12, 60)	0.8096	–0.0050	(26, 130)	0.8184	0.0007
	(5, 50)	0.8715	–0.0050	(8, 80)	0.8166	–0.0139	(19, 190)	0.8245	0.0073
3.5	(9, 9)	0.8429	–0.0033	(14, 14)	0.8020	–0.0015	(23, 23)	0.8090	–0.0036
	(6, 12)	0.8303	0.0053	(10, 20)	0.8215	–0.0056	(16, 32)	0.8142	0.0018
	(5, 25)	0.9609	0.0025	(6, 30)	0.8569	–0.0023	(10, 50)	0.8286	0.0009
	(3, 30)	0.9121	0.0029	(4, 40)	0.8641	–0.0014	(7, 70)	0.8172	0.0000

**Table 7:** The computed sample size, simulated assurance probability and errors for the 95% reference intervals of the distribution proportion 0.90 with assurance probability 0.90.

	One-sided CI *C*_*LP*_ and *C*_*UP*_			Two-sided CI *C*_*MP*_			Two-sided CI *C*_*ET*_		
η	(*N*_1_, *N*_2_)	Simulated assurance probability	Difference	(*N*_1_, *N*_2_)	Simulated assurance probability	Difference	(*N*_1_, *N*_2_)	Simulated assurance probability	Difference
2.5	(43, 43)	0.9045	–0.0012	(432, 432)	0.9040	0.0033	(906, 906)	0.9000	–0.0002
	(30, 60)	0.9070	0.0033	(289, 578)	0.8996	–0.0007	(635, 1270)	0.9056	0.0054
	(19, 95)	0.9122	0.0057	(149, 745)	0.9038	0.0019	(398, 1990)	0.8988	–0.0016
	(14, 140)	0.9094	0.0022	(85, 850)	0.9026	0.0020	(293, 2930)	0.9028	0.0017
3.0	(18, 18)	0.9193	–0.0002	(40, 40)	0.9048	0.0015	(72, 72)	0.8991	–0.0049
	(12, 24)	0.9050	0.0018	(27, 54)	0.9059	0.0037	(50, 100)	0.9020	–0.0006
	(8, 40)	0.9349	0.0009	(15, 75)	0.9133	0.0041	(31, 155)	0.9078	0.0007
	(6, 60)	0.9519	0.0010	(10, 100)	0.9242	–0.0042	(22, 220)	0.9026	0.0003
3.5	(11, 11)	0.9322	0.0010	(18, 18)	0.9192	0.0043	(28, 28)	0.9041	–0.0020
	(8, 16)	0.9475	0.0016	(12, 24)	0.9084	0.0009	(20, 40)	0.9136	–0.0033
	(5, 25)	0.9584	0.0000	(7, 35)	0.9235	–0.0008	(12, 60)	0.9172	0.0006
	(3, 30)	0.9098	0.0006	(5, 50)	0.9589	0.0035	(9, 90)	0.9461	0.0034

In the second stage, simulated values of expected width and assurance probability associated with the reported sample sizes and selected parameter configurations were computed through a Monte Carlo study of 10,000 independent data sets. For each replicate, two groups of *N*_1_ and *N*_2_ values were generated from the normal distribution *N*(0, 1). The sample variance and critical values were obtained to compute the half-width estimates {*H*_*τ*_, *H*_*g*_, *H*_*h*_} for the one- and two-sided confidence intervals {*C*_*LP*_, *C*_*UP*_, *C*_*MP*_, *C*_*ET*_}. The simulated expected half-width is the mean of the 10,000 replicates of the half-width estimates. Also, the simulated assurance probability is the proportion of the 10,000 replicates whose half-width estimates are less than or equal to the specified bound η. The adequacy of the examined sample size procedures for precise interval estimation is determined by the difference between the attained precision quantity and the simulated result. Due to the discrete nature of sample sizes, the obtained half-width of the reported sample sizes are marginally smaller than the designated threshold η. On the other hand, the attained assurance probability is slightly higher than the nominal assurance level 1 –γ. Accordingly, the adequacy of the sample size procedures for precise interval estimation is determined by the difference between the simulated half-width and the computed half-width, and the difference between the simulated assurance probability and the computed assurance probability. These values are also summarized in Tables 2–7 for the expected half-width and assurance probability evaluations.

The prescribed numerical assessments suggest that the required sample sizes in Tables 2–7 vary drastically across the types of reference ranges and precision considerations. In general, under the expected half-width consideration, the optimal sample size in Tables 2 and 3 increases with a smaller width bound of η when all other factors are fixed. For the assurance probability criterion in Tables 4–7, a larger sample size is needed to attain a higher assurance level 1 –γ when the designated width η and other configurations remain identical. With the same settings, the optimal sample size of the one-sided reference ranges *C*_*LP*_ and *C*_*UP*_ is less than those of the two-sided reference ranges. Also, the two-sided reference range *C*_*ET*_ to control equal-tails demands more sample size than the counterpart two-sided *C*_*MP*_ for a major proportion. Under the examined precision settings, the results reveal that the assurance probability principle generally requires larger sample size than the expected half-width criterion as shown in Tables 2, 4 and 5. The extensive evaluations reveal that it is more efficient to adopt a balance design than an unbalance design because the former require less sample size to attain the same precision for the one- and two-sided reference ranges. Regarding the appraisal of sample size determinations, both the simulated half-widths and simulated assurance probabilities are nearly the same as the nominal precision bounds for all cases examined here. The performance of the suggested sample size procedures is sufficiently accurate for most purposes. Accordingly, these sample size formulas are useful for obtaining precise reference ranges under the prescribed expected half-width and assurance probability criteria.

### An example

A randomized parallel group study was conducted in Liu et al. [33] to examine the pharmacokinetic properties and bioequivalence of two sulfadoxine/pyrimethamine fixed-dose combination tablets. The study found that the test and reference formulations of sulfadoxine/pyrimethamine FDC 500/25-mg tablet have similar pharmacokinetic profiles both in terms of rate and extent of absorption. One of the primary endpoints used in the study is the area under the plasma concentration-time curve at 72 hours of pyrimethamine. For the test and reference formulations, the sample means and sample standard deviations are ${\bar{X}}_{1}=3.3898$, ${\bar{X}}_{2}=3.4394$, *S*_1_ = 0.1132, and *S*_2_ = 0.1314 of the *N*_1_ = *N*_2_ = 23 log-transformed measurements. Accordingly, the conventional 95% two-sided confidence interval of mean difference is (–0.1225, 0.0233).

For illustration, numerical examination is extended to compute the reference ranges of the proportion p* = 0.9 of measurement differences between the test and reference formulations. With the suggested algorithms and formulations, the three critical values for a 95% confidence level are τ_0.95_ = 8.3847, *g*_0.95_ = 9.8477, and *h*_0.95_ = 10.9049. The one-sided 95% reference ranges of the 0.9 proportion computed from Eqs 5 and 6 are *C*_*LP*_ = (–∞, 0.2536) and *C*_*UP*_ = (–0.3528, ∞), respectively. The two-sided 95% reference range for a 0.9 proportion without an equal-tail restriction is *C*_*MP*_ = (–0.4057, 0.3065). Whereas the two-sided 95% reference range to control equal-tail proportions is *C*_*ET*_ = (–0.4440, 0.3448). These reference ranges for distribution proportion of measurement differences are evidently much wider than the usual confidence interval of a single parameter of mean difference.

Moreover, these model settings are employed to explicate precision assessments and sample size calculations for future comparative study. With σ = 0.122638, p* = 0.9, and 1 –α = 0.95, the necessary sample sizes for the interval procedures *C*_*LP*_, *C*_*MP*_, and *C*_*ET*_ to have an expected half-width η = 0.3 are (*N*_1_, *N*_2_) = (24, 24), (301, 301), and (929, 929), respectively. The corresponding sample sizes for attaining the assurance probability 1 –γ = 0.9 are (40, 40), (640, 640), and (1508, 1508) for the three reference ranges *C*_*LP*_, *C*_*MP*_, and *C*_*ET*_. Notably, the influence of expected half-width and assurance probability principles on sample size requirements not only differs but also depends on the types of reference range procedures. The presented sample size formulas and accompany computer algorithms can be readily used to assess the nonlinear relationship between the key features. For ease of application, the exemplifying configurations are incorporated in the user specification sections of the supplemental computer programs. Users can easily modify the exemplifying settings in the programs to accommodate their own model specifications.

## Conclusions

Reference ranges are constructed to comprise a desired large proportion of the population measurements and provide the potential spread of the target probability distribution. With the connection between reference range and tolerance interval procedures, the technical features of reference ranges can be readily obtained from the statistical literature for the estimation of tolerance intervals. This study addresses two important aspects to complement the existing appraisals and demonstrations. First, a complete collection of reference ranges is presented within the context of parallel group designs under variance homogeneity assumption. They include one- and two-sided reference ranges to control the major proportion of a distribution and two-sided reference ranges to control equal tail probability of a distribution. Second, sample size determinations for precise reference ranges are considered under the expected half-width and assurance probability principles. Analytical formulas and computer algorithms are described to extend the practical value of reference ranges for parallel group studies. Their usefulness and accuracy are illustrated and justified through empirical illustrations and simulation assessments. The suggested procedures are also demonstrated with a real data regarding the comparison of tablet absorption rate and extent between two drug formulations.

## Supporting information

S1 File Appendix A: The critical values of two-sided reference ranges. (PDF)

S2 File SAS/IML programs for performing the suggested procedures. (PDF)

S3 File R programs for performing the suggested procedures. (PDF)
