# Supplementary material for: Determining reference ranges and sample sizes in parallel-group studies
Source: PLoS One. 2022 Nov 30;17(11):e0278447. doi: 10.1371/journal.pone.0278447 (PMC9710766; doi:10.1371/journal.pone.0278447)
Supplement: S1 File — (PDF) [file pone.0278447.s001.pdf]

Appendix A. The critical values of two-sided reference ranges

A1. The critical values of two-sided reference ranges for a major proportion

It follows from the normal assumption defined in Equation 1 that

$$Z = (\bar{X}_1 - \bar{X}_2 - \mu_D)/(\sigma^2/M)^{1/2} \sim N(0, 1) \text{ and } K = \nu S^2/\sigma^2 \sim \chi^2(\nu) \quad \text{(A1)}$$

where $\chi^2(\nu)$ is a chi-square distribution with degrees of freedom ν. Also, Z and K are independent. Accordingly, the inequality $T_{MPL} < D < T_{MPU}$ can be rewritten as $-g_{1-\alpha} < \{(2M\nu)/K\}^{1/2}Z^* < g_{1-\alpha}$, where $Z^* = (D - \mu_D)/\sigma_D - Z/(2M)^{1/2}$ and $(D - \mu_D)/\sigma_D \sim N(0, 1)$. For given values of $(\bar{X}_1 - \bar{X}_2, S^2)$ or (Z, K), $Z^* \sim N(-Z/(2M)^{1/2}, 1)$ and $K^* = Z^{*2} \sim \chi^2(1, \lambda^2)$ where $\chi^2(1, \lambda^2)$ is a noncentral chi-square distribution with one degree of freedom and noncentrality $\lambda^2 = Z^2/(2M)$. It follows that $P[T_{MPL} < D < T_{MPU}|(\bar{X}_1 - \bar{X}_2, S^2)] \geq p^*$ is equivalent to $P[\{(2M\nu)/K\}K^* < g^2_{1-\alpha}|(\bar{X}_1 - \bar{X}_2, S^2)] \geq p^*$ or $P[K^* < (g^2_{1-\alpha}K)/(2M\nu)|(\bar{X}_1 - \bar{X}_2, S^2)] \geq p^*$. This suggests that $\chi^2_{p^*}(1, \lambda^2) < (g^2_{1-\alpha}K)/(2M\nu)$ where $\chi^2_{p^*}(1, \lambda^2)$ is the $(100 \cdot p^*)$th percentile of $\chi^2(1, \lambda^2)$. Therefore, Equation 8 can be expressed as

$$P\{K > K_g\} = 1 - \alpha, \quad \text{(A2)}$$

where $K_g = (2M\nu)\chi^2_{p^*}(1, \lambda^2)/g^2_{1-\alpha}$. Let $\Phi_K(\cdot)$ be the cumulative distribution function of the chi-square random variable $K \sim \chi^2(\nu)$. The probability assessment implies that the critical value $g_{1-\alpha}$ can be uniquely determined from

$$E_Z\{1 - \Phi_K(K_g)\} = 1 - \alpha, \quad \text{(A3)}$$

where the expectation E_Z is taken with respect to the standard normal distribution Z.

A2. The critical values of two-sided reference ranges for the central proportion

With the prescribed variables Z and K in Equation A1, it follows that the inequalities $T_{ETL} < \theta_{1-p}$ and $\theta_p < T_{ETU}$ lead to the conditions of $(K/\nu)^{1/2} > [Z + (2M)^{1/2}z_p]/h_{1-\alpha}$ and $(K/\nu)^{1/2} > [-Z$

$+ (2M)^{1/2}z_p]/h_{1-\alpha}$, respectively. The joint consideration of the two conditions implies that $(K/\nu)^{1/2} > [|Z| + (2M)^{1/2}z_p]/h_{1-\alpha}$ and Equation 11 can be written as

$$P\{K > K_h\} = 1 - \alpha, \tag{A4}$$

where $K_h = (2M\nu)\{|Z|/(2M)^{1/2} + z_p\}^2/h^2_{1-\alpha}$. The confidence level assures that the critical value $h_{1-\alpha}$ can be uniquely computed from

$$E_Z\{1 - \Phi_K(K_h)\} = 1 - \alpha, \tag{A5}$$

where the expectation E_Z is taken with respect to the standard normal distribution Z.
