# Supplementary material for: Determining reference ranges and sample sizes in parallel-group studies
Source: PLoS One. 2022 Nov 30;17(11):e0278447. doi: 10.1371/journal.pone.0278447 (PMC9710766; doi:10.1371/journal.pone.0278447)
Supplement: S2 File — (PDF) [file pone.0278447.s002.pdf]

Appendix S1

SAS/IML program for computing the reference ranges of the designated proportion

```
PROC IML;
*USER SPECIFICATION PORTION;
*DESIGNATED ALPHA;ALPHA=0.05;
*SAMPLE SIZES;N1=23;N2=23;
*MEAN DIFFERENCE;MED=-0.0496;
*SAMPLE STANDARD DEVIATION;S=0.122638;
*PROPORTION;PROP=0.90;
*END OF USER SPECIFICATION PORTION;

CP=1-ALPHA;PCT=1-(1-PROP)/2;
ZPCT=QUANTILE('NORMAL',PCT);ZPROP=QUANTILE('NORMAL',PROP);
DF=N1+N2-2;M=1/(1/N1+1/N2);SQRM=SQRT(M);
PRINT ALPHA PROP PCT N1 N2 MED S ZPROP[FORMAT=8.4] ZPCT[FORMAT=8.4];

*ONE-SIDED CI;
TAU=QUANTILE('T',1-ALPHA,DF,ZPROP#SQRT(2#M));
TLPU=MED+TAU#S/SQRM;
TUPL=MED-TAU#S/SQRM;
PRINT 'ONE-SIDED REFERENCE RANGE: CLP' TAU[FORMAT=8.4] '  -INF  '
TLPU[FORMAT=8.4];
PRINT 'ONE-SIDED REFERENCE RANGE: CUP' TAU[FORMAT=8.4]
TUPL[FORMAT=8.4] '  +INF  ';

NUMINT=1000;LC=NUMINT+1;
COEVEC=({1}||REPEAT({4 2},1,NUMINT/2-1)||{4 1})`;
ZQL=QUANTILE('NORMAL',10E-8);ZQU=-ZQL;
INT=ZQU-ZQL;INTL=INT/NUMINT;
ZVEC=ZQL+(INTL#(0:NUMINT))`;
WZPDF=(INTL/3)#COEVEC#PDF('NORMAL',ZVEC);

START GFUN;
GL=0;GU=100;
DO UNTIL(ABS(DD)<10E-9 & DD>0);
GT=(GL+GU)/2;
KG=SQRT(QUANTILE('CHISQ',PROP,1,ZVEC##2/(2#M)));
CPG=WZPDF`*(1-CDF('CHISQ',((DF#2#M)/(GT##2))#KG##2,DF));
IF CPG>CP THEN GU=GT;ELSE GL=GT;
DD=CPG-CP;END;
FINISH;

START HFUN;
HL=0;HU=100;
DO UNTIL(ABS(DD)<10E-9 & DD>0);
HT=(HL+HU)/2;
KH=QUANTILE('NORMAL',PCT,ABS(ZVEC)/SQRT(2#M),1);
CPH=WZPDF`*(1-CDF('CHISQ',((DF#2#M)/(HT##2))#KH##2,DF));
IF CPH>CP THEN HU=HT;ELSE HL=HT;
DD=CPH-CP;END;
```

```
FINISH;

RUN GFUN;G=GT;
TMPL=MED-G#S/SQRM;TMPU=MED+G#S/SQRM;
PRINT 'MAJOR PROPORTION REFERENCE RANGE';
PRINT G[FORMAT=8.4] TMPL[FORMAT=8.4] TMPU[FORMAT=8.4];
RUN HFUN;H=HT;
TETL=MED-H#S/SQRM;TETU=MED+H#S/SQRM;
PRINT 'EQUAL TAILS REFERENCE RANGE';
PRINT H[FORMAT=8.4] TETL[FORMAT=8.4] TETU[FORMAT=8.4];
QUIT;
```

Appendix S2

SAS/IML program for computing sample size required to meet the expected half-width for reference ranges of the designated proportion

```
PROC IML;
*USER SPECIFICATION PORTION;
*DESIGNATED ALPHA;ALPHA=0.05;
*SAMPLE SIZE RATIO;R21=1;
*EXPECTED HALF-WIDTH;ETA=0.3;
*MEAN DIFFERENCE;MUD=-0.0496;
*STANDARD DEVIATION;SIGMA=0.122638;
*PROPORTION;PROP=0.90;
*END OF USER SPECIFICATION PORTION;

CP=1-ALPHA;PCT=1-(1-PROP)/2;
ZPCT=QUANTILE('NORMAL',PCT);ZPROP=QUANTILE('NORMAL',PROP);
PRINT ALPHA PROP PCT MUD SIGMA ZPROP[FORMAT=8.4] ZPCT[FORMAT=8.4];

SIGSQ=SIGMA##2;SIGSQD=2#SIGSQ;SIGMAD=SQRT(SIGSQD);
THETAL=MUD-ZPCT#SIGMAD;THETAU=MUD+ZPCT#SIGMAD;
THETAPROPL=MUD-ZPROP#SIGMAD;THETAPROPU=MUD+ZPROP#SIGMAD;
PRINT THETAPROPL[FORMAT=8.4] THETAPROPU[FORMAT=8.4]
THETAL[FORMAT=8.4] THETAU[FORMAT=8.4];

NUMINT=200;LC=NUMINT+1;
COEVEC=({1}||REPEAT({4 2},1,NUMINT/2-1)||{4 1})`;
ZQL=QUANTILE('NORMAL',10E-8);ZQU=-ZQL;
INT=ZQU-ZQL;INTL=INT/NUMINT;
ZVEC=ZQL+(INTL#(0:NUMINT))`;
WZPDF=(INTL/3)#COEVEC#PDF('NORMAL',ZVEC);

START GFUN;
GL=0;GU=100;
DO UNTIL(ABS(DD)<10E-5 & DD>0);
GT=(GL+GU)/2;
KG=SQRT(QUANTILE('CHISQ',PROP,1,ZVEC##2/(2#M)));
CPG=WZPDF`*(1-CDF('CHISQ',((DF#2#M)/(GT##2))#KG##2,DF));
IF CPG>CP THEN GU=GT;ELSE GL=GT;
DD=CPG-CP;END;
FINISH;

START HFUN;
HL=0;HU=100;
DO UNTIL(ABS(DD)<10E-5 & DD>0);
HT=(HL+HU)/2;
KH=QUANTILE('NORMAL',PCT,ABS(ZVEC)/SQRT(2#M),1);
CPH=WZPDF`*(1-CDF('CHISQ',((DF#2#M)/(HT##2))#KH##2,DF));
IF CPH>CP THEN HU=HT;ELSE HL=HT;
DD=CPH-CP;END;
FINISH;
```

```
START EWNTAU;
N1=4;
DO UNTIL(ETATAU<ETA | N1>2000);
N1=N1+1;N2=N1#R21;DF=N1+N2-2;M=1/(1/N1+1/N2);
LOGU=LOG(SQRT(DF/2))+LGAMMA(DF/2)-LGAMMA((DF+1)/2);U=EXP(LOGU)
;
TAU=QUANTILE('T',1-ALPHA,DF,ZPROP#SQRT(2#M));
ETATAU=TAU#SIGMA/(U#SQRT(M));
END;TAUN1=N1;TAUN2=N2;
PRINT 'ONE-SIDED REFERENCE RANGE';
PRINT ETA ETATAU[FORMAT=8.4] TAU[FORMAT=8.4] TAUN1 TAUN2;
FINISH;
RUN EWNTAU;

START EWNG;
N1=4;
DO UNTIL(ETAG<ETA | N1>2000);
N1=N1+1;N2=N1#R21;DF=N1+N2-2;M=1/(1/N1+1/N2);
LOGU=LOG(SQRT(DF/2))+LGAMMA(DF/2)-LGAMMA((DF+1)/2);U=EXP(LOGU)
;
RUN GFUN;G=GT;
ETAG=G#SIGMA/(U#SQRT(M));
END;GN1=N1;GN2=N2;
PRINT 'MAJOR PROPORTION REFERENCE RANGE';
PRINT ETA ETAG[FORMAT=8.4] G[FORMAT=8.4] GN1 GN2;
FINISH;
RUN EWNG;

START EWNH;
N1=4;
DO UNTIL(ETAH<ETA | N1>2000);
N1=N1+1;N2=N1#R21;DF=N1+N2-2;M=1/(1/N1+1/N2);
LOGU=LOG(SQRT(DF/2))+LGAMMA(DF/2)-LGAMMA((DF+1)/2);U=EXP(LOGU)
;
RUN HFUN;H=HT;
ETAH=H#SIGMA/(U#SQRT(M));
END;HN1=N1;HN2=N2;
PRINT 'EQUAL TAILS REFERENCE RANGE';
PRINT ETA ETAH[FORMAT=8.4] H[FORMAT=8.4] HN1 HN2;
FINISH;
RUN EWNH;
QUIT;
```

Appendix S3

SAS/IML program for computing sample size required to ensure the assurance probability for reference ranges of the designated proportion

```
PROC IML;
*USER SPECIFICATION PORTION;
*DESIGNATED ALPHA;ALPHA=0.05;
*SAMPLE SIZE RATIO;R21=1;
*EXPECTED HALF-WIDTH;ETA=0.3;
*ASSURANCE PROBABILITY;ASP=0.8;
*MEAN DIFFERENCE;MUD=-0.0496;
*STANDARD DEVIATION;SIGMA=0.122638;
*PROPORTION;PROP=0.90;
*END OF USER SPECIFICATION PORTION;

CP=1-ALPHA;PCT=1-(1-PROP)/2;
ZPCT=QUANTILE('NORMAL',PCT);ZPROP=QUANTILE('NORMAL',PROP);
PRINT ALPHA PROP PCT MUD SIGMA ZPROP[FORMAT=8.4] ZPCT[FORMAT=8.4];

SIGSQ=SIGMA##2;SIGSQD=2#SIGSQ;SIGMAD=SQRT(SIGSQD);
THETAL=MUD-ZPCT#SIGMAD;THETAU=MUD+ZPCT#SIGMAD;
THETAPROPL=MUD-ZPROP#SIGMAD;THETAPROPU=MUD+ZPROP#SIGMAD;
PRINT THETAPROPL[FORMAT=8.4] THETAPROPU[FORMAT=8.4]
THETAL[FORMAT=8.4] THETAU[FORMAT=8.4];

NUMINT=200;LC=NUMINT+1;
COEVEC=({1}||REPEAT({4 2},1,NUMINT/2-1)||{4 1})`;
ZQL=QUANTILE('NORMAL',10E-8);ZQU=-ZQL;
INT=ZQU-ZQL;INTL=INT/NUMINT;
ZVEC=ZQL+(INTL#(0:NUMINT))`;
WZPDF=(INTL/3)#COEVEC#PDF('NORMAL',ZVEC);

START GFUN;
GL=0;GU=100;
DO UNTIL(ABS(DD)<10E-5 & DD>0);
GT=(GL+GU)/2;
KG=SQRT(QUANTILE('CHISQ',PROP,1,ZVEC##2/(2#M)));
CPG=WZPDF`*(1-CDF('CHISQ',((DF#2#M)/(GT##2))#KG##2,DF));
IF CPG>CP THEN GU=GT;ELSE GL=GT;
DD=CPG-CP;END;
FINISH;

START HFUN;
HL=0;HU=100;
DO UNTIL(ABS(DD)<10E-5 & DD>0);
HT=(HL+HU)/2;
KH=QUANTILE('NORMAL',PCT,ABS(ZVEC)/SQRT(2#M),1);
CPH=WZPDF`*(1-CDF('CHISQ',((DF#2#M)/(HT##2))#KH##2,DF));
IF CPH>CP THEN HU=HT;ELSE HL=HT;
DD=CPH-CP;END;
FINISH;
```

```
START APNTAU;
N1=4;
DO UNTIL(ASPTAU>ASP | N1>2000);
N1=N1+1;N2=N1#R21;DF=N1+N2-2;M=1/(1/N1+1/N2);
TAU=QUANTILE('T',1-ALPHA,DF,ZPROP#SQRT(2#M));
ASPTAU=CDF('CHISQ',(DF#M#ETA##2)/(TAU#SIGMA)##2,DF);
END;TAUN1=N1;TAUN2=N2;
PRINT 'ONE-SIDED REFERENCE RANGE';
PRINT ASP ASPTAU[FORMAT=8.4] TAU[FORMAT=8.4] TAUN1 TAUN2;
FINISH;
RUN APNTAU;

START APNG;
N1=4;
DO UNTIL(ASPG>ASP | N1>2000);
N1=N1+1;N2=N1#R21;DF=N1+N2-2;M=1/(1/N1+1/N2);
RUN GFUN;G=GT;
ASPG=CDF('CHISQ',(DF#M#ETA##2)/(G#SIGMA)##2,DF);
END;GN1=N1;GN2=N2;
PRINT 'MAJOR PROPORTION REFERENCE RANGE';
PRINT ASP ASPG[FORMAT=8.4] G[FORMAT=8.4] GN1 GN2;
FINISH;
RUN APNG;

START APNH;
N1=4;
DO UNTIL(ASPH>ASP | N1>2000);
N1=N1+1;N2=N1#R21;DF=N1+N2-2;M=1/(1/N1+1/N2);
RUN HFUN;H=HT;
ASPH=CDF('CHISQ',(DF#M#ETA##2)/(H#SIGMA)##2,DF);
END;HN1=N1;HN2=N2;
PRINT 'EQUAL TAILS REFERENCE RANGE';
PRINT ASP ASPH[FORMAT=8.4] H[FORMAT=8.4] HN1 HN2;
FINISH;
RUN APNH;
QUIT;
```
