# Supplementary material for: Determining reference ranges and sample sizes in parallel-group studies
Source: PLoS One. 2022 Nov 30;17(11):e0278447. doi: 10.1371/journal.pone.0278447 (PMC9710766; doi:10.1371/journal.pone.0278447)
Supplement: S3 File — (PDF) [file pone.0278447.s003.pdf]

Appendix R1

R program for computing the reference ranges of the designated proportion

```
function () {
#USER SPECIFICATIONS PORTION
alpha=0.05 #DESIGNATED ALPHA
n1=23
n2=23 #SAMPLE SIZES
med=-0.0496 #SAMPLE MEAN DIFFERENCE
s=0.122638 #SAMPLE STANDARD DEVIATION
prop=0.90 #PROPORTION
#END OF SPECIFICATION

cp=1-alpha
pct=(1+prop)/2
zpct=qnorm(pct)
zprop=qnorm(prop)
df=n1+n1-2
m=1/(1/n1+1/n2)
sqrm=sqrt(m)
print(c("alpha, prop, pct, n1, n2, zprop, zpct"))
print(c(alpha,prop,pct,n1,n2,zprop,zpct))

tau=qt(1-alpha,df,zprop*sqrt(2*m))
tlpu=med+tau*s/sqrm
tupl=med-tau*s/sqrm
print("ONE-SIDED REFERENCE RANGE CLP: tau, -inf, tlpu")
print(c(tau, -Inf, tlpu))
print("ONE-SIDED REFERENCE RANGE CUP: tau, tpl, inf")
print(c(tau, tupl, Inf))

numint=1000
coevec=c(1,rep(c(4,2),numint/2-1),4,1)
zql=qnorm(1e-8)
zqu=-zql
int=zqu-zql
intl=int/numint
zvec=zql+intl*(0:numint)
wzpdf=(intl/3)*coevec*dnorm(zvec)

gfun=function(){
gl=0
gu=100
dd=1
while(abs(dd)>1e-9 | dd<0){
gt=(gl+gu)/2
kg=sqrt(qchisq(prop,1,zvec^2/(2*m)))
cpg=sum(wzpdf*(1-pchisq(((df*2*m)/gt^2)*kg^2,df)))
if (cpg>cp) gu=gt else gl=gt
dd=cpg-cp}
return(gt)
}
```

```
hfun=function(){
hl=0
hu=100
dd=1
while(abs(dd)>1e-9 | dd<0){
ht=(hl+hu)/2
kh=qnorm(pct,abs(zvec)/sqrt(2*m),1)
cph=sum(wzpdf*(1-pchisq(((df*2*m)/ht^2)*kh^2,df)))
if (cph>cp) hu=ht else hl=ht
dd=cph-cp}
return(ht)
}
g=gfun()
tmpl=med-g*s/sqrm
tmpu=med+g*s/sqrm
h=hfun()
tetl=med-h*s/sqrm
tetu=med+h*s/sqrm
print("MAJOR PROPORTION REFERENCE RANGE: g, tmpl, tmpu")
print(c(g,tmpl,tmpu))
print("EQUAL TAILS REFERENCE RANGE: h, tetl, tetu")
print(c(h,tetl,tetu))
}
```

Appendix R2

R program for computing sample size required to meet the expected half-width for reference ranges of the designated proportion

```
function () {
#USER SPECIFICATIONS PORTION
alpha=0.05 #DESIGNATED ALPHA
r21=1 #SAMPLE SIZE RATIO
eta=0.3 #EXPECTED HALF-WIDTH
mud=-0.0496 #MEAN DIFFERENCE
sigma=0.122638 #STANDARD DEVIATION
prop=0.90 #PROPORTION
#END OF SPECIFICATION

cp=1-alpha
pct=(1+prop)/2
zpct=qnorm(pct)
zprop=qnorm(prop)
print(c("alpha, prop, pct, mud, sigma, zprop, zpct"))
print(c(alpha,prop,pct,mud,sigma,zprop,zpct))

sigsq=sigma^2
sigsqd=2*sigsq
sigmad=sqrt(sigsqd)
thetal=mud-zpct*sigmad
thetau=mud+zpct*sigmad
thetapropl=mud-zprop*sigmad
thetapropu=mud+zprop*sigmad
print("thetapropl, thetapropu, thetal, thetau")
print(c(thetapropl,thetapropu,thetal,thetau))

numint=200
coevec=c(1,rep(c(4,2),numint/2-1),4,1)
zql=qnorm(1e-8)
zqu=-zql
int=zqu-zql
intl=int/numint
zvec=zql+intl*(0:numint)
wzpdf=(intl/3)*coevec*dnorm(zvec)

gfun=function(){
gl=0
gu=100
dd=1
while(abs(dd)>1e-5 | dd<0){
gt=(gl+gu)/2
kg=sqrt(qchisq(prop,1,zvec^2/(2*m)))
cpg=sum(wzpdf*(1-pchisq(((df*2*m)/gt^2)*kg^2,df)))
if (cpg>cp) gu=gt else gl=gt
dd=cpg-cp}
return(gt)
}
```

```
hfun=function(){
hl=0
hu=100
dd=1
while(abs(dd)>1e-5 | dd<0){
ht=(hl+hu)/2
kh=qnorm(pct,abs(zvec)/sqrt(2*m),1)
cph=sum(wzpdf*(1-pchisq(((df*2*m)/ht^2)*kh^2,df)))
if (cph>cp) hu=ht else hl=ht
dd=cph-cp}
return(ht)
}

n1=4
etatau=100
while(etatau>eta & n1<2000){
n1=n1+1
n2=r21*n1
df=n1+n1-2
m=1/(1/n1+1/n2)
logu<-log(sqrt(df/2))+lgamma(df/2)-lgamma((df+1)/2)
u<-exp(logu)
tau=qt(1-alpha,df,zprop*sqrt(2*m))
etatau=tau*sigma/(u*sqrt(m))}
taun1=n1
taun2=n2
print("ONE-SIDED REFERENCE RANGE: eta, etatau, tau, taun1, taun2")
print(c(eta,etatau,tau,taun1,taun2))

n1=4
etag=100
while(etag>eta & n1<2000){
n1=n1+1
n2=r21*n1
df=n1+n1-2
m=1/(1/n1+1/n2)
logu<-log(sqrt(df/2))+lgamma(df/2)-lgamma((df+1)/2)
u<-exp(logu)
g=gfun()
etag=g*sigma/(u*sqrt(m))}
gn1=n1
gn2=n2
print("MAJOR PROPORTION REFERENCE RANGE: eta, etag, g, gn1, gn2")
print(c(eta,etag,g,gn1,gn2))

n1=4
etah=100
while(etah>eta & n1<2000){
n1=n1+1
n2=r21*n1
df=n1+n1-2
```

```
m=1/(1/n1+1/n2)
logu<-log(sqrt(df/2))+lgamma(df/2)-lgamma((df+1)/2)
u<-exp(logu)
h=hfun()
etah=h*sigma/(u*sqrt(m))}
hn1=n1
hn2=n2
print("EQUAL TAILS REFERENCE RANGE: eta, etah, h, hn1, hn2")
print(c(eta,etah,h,hn1,hn2))
}
```

Appendix R3

R program for computing sample size required to ensure the assurance probability for reference ranges of the designated proportion

```
function () {
#USER SPECIFICATIONS PORTION
alpha=0.05 #DESIGNATED ALPHA
r21=1 #SAMPLE SIZE RATIO
eta=0.3 #EXPECTED HALF-WIDTH
asp=0.8 #ASSURANCE PROBABILITY
mud=-0.0496 #MEAN DIFFERENCE
sigma=0.122638 #STANDARD DEVIATION
prop=0.90 #PROPORTION
#END OF SPECIFICATION

cp=1-alpha
pct=(1+prop)/2
zpct=qnorm(pct)
zprop=qnorm(prop)
print(c("alpha, prop, pct, mud, sigma, zprop, zpct"))
print(c(alpha,prop,pct,mud,sigma,zprop,zpct))

sigsq=sigma^2
sigsqd=2*sigsq
sigmad=sqrt(sigsqd)
thetal=mud-zpct*sigmad
thetau=mud+zpct*sigmad
thetapropl=mud-zprop*sigmad
thetapropu=mud+zprop*sigmad
print("thetapropl, thetapropu, thetal, thetau")
print(c(thetapropl,thetapropu,thetal,thetau))

numint=200
coevec=c(1,rep(c(4,2),numint/2-1),4,1)
zql=qnorm(1e-8)
zqu=-zql
int=zqu-zql
intl=int/numint
zvec=zql+intl*(0:numint)
wzpdf=(intl/3)*coevec*dnorm(zvec)

gfun=function(){
gl=0
gu=100
dd=1
while(abs(dd)>1e-4 | dd<0){
gt=(gl+gu)/2
kg=sqrt(qchisq(prop,1,zvec^2/(2*m)))
cpg=sum(wzpdf*(1-pchisq(((df*2*m)/gt^2)*kg^2,df)))
if (cpg>cp) gu=gt else gl=gt
dd=cpg-cp}
```

```
return(gt)
}

hfun=function(){
hl=0
hu=100
dd=1
while(abs(dd)>1e-4 | dd<0){
ht=(hl+hu)/2
kh=qnorm(pct,abs(zvec)/sqrt(2*m),1)
cph=sum(wzpdf*(1-pchisq(((df*2*m)/ht^2)*kh^2,df)))
if (cph>cp) hu=ht else hl=ht
dd=cph-cp}
return(ht)
}

n1=4
asptau=0
while(asptau<asp & n1<2000){
n1=n1+1
n2=r21*n1
df=n1+n1-2
m=1/(1/n1+1/n2)
tau=qt(1-alpha,df,zprop*sqrt(2*m))
asptau=pchisq((df*m*eta^2)/(tau*sigma)^2,df)}
taun1=n1
taun2=n2
print("ONE-SIDED REFERENCE RANGE: asp, asptau, tau, taun1, taun2")
print(c(asp,asptau,tau,taun1,taun2))

n1=4
aspg=0
while(aspg<asp & n1<2000){
n1=n1+1
n2=r21*n1
df=n1+n1-2
m=1/(1/n1+1/n2)
g=gfun()
aspg=pchisq((df*m*eta^2)/(g*sigma)^2,df)}
gn1=n1
gn2=n2
print("MAJOR PROPORTION REFERENCE RANGE: asp, aspg, g, gn1, gn2")
print(c(asp,aspg,g,gn1,gn2))

n1=4
asph=0
while(asph<asp & n1<2000){
n1=n1+1
n2=r21*n1
df=n1+n1-2
m=1/(1/n1+1/n2)
h=hfun()
```

```
asph=pchisq((df*m*eta^2)/(h*sigma)^2,df)}
hn1=n1
hn2=n2
print("EQUAL TAILS REFERENCE RANGE: asp, asph, h, hn1, hn2")
print(c(asp,asph,h,hn1,hn2))
}
```
